# Cardio-Oncology in Older Adults: Evidence Gaps, Geriatric Considerations, and Future Directions

**DOI:** 10.3390/jcm15176592

**Published:** 2026-08-26

**Authors:** Vasiliki Katsi, Lefki Nikolopoulou, Christos Fragoulis, Vasilios Kordalis, Konstantinos Tsioufis, Konstantinos Toutouzas

**Affiliations:** 1 First Department of Cardiology, Hippokration General Hospital, 11527 Athens, Greece; vkkatsi@yahoo.gr (V.K.); christosfragoulis@yahoo.com (C.F.); kordalisvg@gmail.com (V.K.); ktsioufis@gmail.com (K.T.); 2 Cardio-Oncology Unit, First Department of Cardiology, Hippokration General Hospital, 11527 Athens, Greece; ktoutouz@gmail.com

**Keywords:** older adults, elderly, cardio-oncology, cardiotoxicity, cancer-treatment

## Abstract

Cardio-oncology has emerged as a critical discipline addressing cardiovascular toxicities from cancer therapies, yet substantial evidence gaps persist for elderly patients, who represent over 60% of new cancer diagnoses. This narrative review synthesizes the current literature on cardio-oncology challenges in older adults (≥65 years), highlighting underrepresentation in clinical trials, compounded risks from frailty, multimorbidity, and polypharmacy, and the paucity of geriatric-specific guidelines. Older adults exhibit heightened susceptibility to cardiotoxicity from antineoplastic therapies due to age-related declines in cardiac reserve and renal function, and baseline atherosclerosis. Comprehensive geriatric assessments (CGA) identify frailty as a key predictor of adverse outcomes, yet their integration into cardio-oncology protocols remains inconsistent, with limited prospective data on risk-stratification tools such as echocardiography and biomarkers. Observational studies underscore disparities in surveillance and management, leading to the undertreatment of cancer and overtreatment of comorbidities. Current cardio-oncology guidelines recognize older age, multimorbidity, frailty, and individualized assessment as important considerations in clinical decision-making. However, validated geriatric-specific thresholds, surveillance strategies, and management algorithms remain limited, particularly for frail older adults and the oldest-old populations. Emerging priorities include multidisciplinary models incorporating geriatricians, randomized trials in frail populations, and real-world evidence on novel agents. This review advocates tailored strategies to optimize shared decision-making, enhance equity, and address current knowledge gaps through CGA-driven research, while proposing a pragmatic age- and frailty-stratified framework to support individualized cardiovascular assessment in elderly cancer patients.

## 1. Introduction

Cardio-oncology is an evolving field focused on the prevention, detection, and management of cardiovascular disease (CVD) and the complications associated with cancer and its treatment, aiming to optimize both oncological and cardiovascular outcomes, as well as the overall health of the cancer patients [1,2]. The global cancer population is represented mainly by older adults, with more than half of all new cancer diagnoses and the majority of cancer-related deaths globally occurring in patients over the age of 65 years [3,4]. Population aging is expected to be accelerated over the next decades, further amplifying the association between cancer and CVD [4,5].

The prevalence of cardiovascular comorbidities increases with advancing age. In older adults, a reduction in physiological reserve has been confirmed, rendering older adults particularly vulnerable and increasing susceptibility to treatment-related cardiotoxicity [3,6,7]. The documented multimorbidity and frailty, especially in elderly cancer populations, increase the cardiovascular risk and heighten the possibility of adverse cardio-oncology outcomes [3,7]. Notwithstanding this, older adults remain significantly underrepresented in clinical trials that form the cardio-oncology guideline-based management, and their exclusion is often based not only on the chronological age, but also on factors such as functional status and comorbidity burden [8,9,10]. This underrepresentation limits the external validity of evidence and contributes to uncertainty in clinical decision-making for this heterogeneous population [8,9,10].

Although previous reviews and consensus documents have described cardiotoxicity associated with anticancer therapies in older patients, important gaps remain regarding how age-related biological changes, frailty, multimorbidity, and functional vulnerability influence cardiovascular risk assessment and treatment decisions [3,6,10]. Most available evidence remains derived from selected clinical trial populations, while real-world elderly patients frequently present with multiple competing risks, reduced physiological reserve, and heterogeneous functional status [8,9,10]. This review, rather than providing another drug-specific summary of cardiotoxicity, critically evaluates the current evidence from a geriatric perspective, focusing on the limitations related to frailty, multimorbidity, biological aging, and the underrepresentation of older adults in clinical trials. In addition, we propose a pragmatic geriatric-integrated framework that brings together cardiovascular risk assessment, CGA, frailty, functional status, and patient-centered considerations to support individualized clinical decision-making in older adults receiving cancer therapy.

## 2. Methods

This narrative review was based on a non-systematic search of PubMed, supplemented by the manual screening of reference lists from relevant articles and key society statements, including those from the European Society of Cardiology (ESC), the International Cardio-Oncology Society (ICOS), and American Heart Association (AHA). The research was performed from January 2026 to July 2026, and the literature search primarily focused on publications from the last decade, from 2015 to 2025, in order to provide a contemporary overview of the field. However, earlier landmark and seminal studies were additionally included when considered essential for understanding the historical evolution, pathophysiological basis, and foundational evidence of cardio-oncology. Search terms included combinations of “cardio-oncology”, “elderly”, “older adults”, “cardiotoxicity”, “frailty”, “multimorbidity”, “comprehensive geriatric assessment”, “functional status”, and “cancer survivorship”, combined with terms related to major anticancer therapies, including “anthracyclines”, “HER2-targeted therapy”, and “immune checkpoint inhibitors”.

We included clinical trials, observational studies, meta-analyses, and contemporary guidelines addressing cardiovascular complications of cancer therapies in older populations. Priority was given to studies involving patients aged ≥65 years, studies reporting age-specific analyses, and studies incorporating geriatric concepts such as frailty, multimorbidity, or functional status. Studies without age-specific analyses were also included when considered essential for understanding the evidence base and current cardio-oncology practice. Case reports were excluded. For descriptive purposes, the available evidence was discussed according to the widely used gerontological age strata of 65–74 years (young-old), 75–84 years (old-old), and ≥85 years (oldest-old) [11].

Studies included in the evidence tables were selected to illustrate the available evidence and its limitations in older adults across the extensively studied therapeutic classes of anthracyclines, HER2-targeted therapies, and immune checkpoint inhibitors (ICIs). Priority was given to landmark studies, large observational cohorts, and studies providing clinically relevant information on age, cardiovascular toxicity, or the representation of older adults.

Artificial intelligence (AI)-assisted tools (OpenAI, San Francisco, CA, USA; version accessed January–July 2026) were used to support literature identification and the generation of illustrative figures. All references identified with AI assistance were independently verified against the original publications by the authors. Study selection, critical appraisal, interpretation of the evidence, and final manuscript preparation remained the responsibility of the authors. The scientific content, interpretation of the evidence, conclusions, and final manuscript were developed exclusively by the authors, who take full responsibility for the accuracy and integrity of the work.

## 3. Conceptual Framework of Aging in Cardio-Oncology

Advances in cancer diagnosis and treatment have significantly improved survival, but, at the same time have led to an increased incidence and recognition of cancer-treatment-related cardiovascular toxicity (CTR-CVT). CTR-CVT is a major concern in cardio-oncology, but its clinical expression and consequences may differ substantially in older adults [1,2]. Age-related changes may amplify susceptibility to myocardial injury from anthracyclines, human epidermal growth factor receptor 2 (HER2) -targeted therapies, ICIs, radiotherapy, and other antineoplastic therapies [12,13,14,15,16,17,18,19,20,21,22,23]. Therefore, understanding the mechanism of cardiotoxicity and applying it to older adults is important and requires the consideration of biological aging rather than chronological age alone [1,3,6,10].

**Figure 1:**
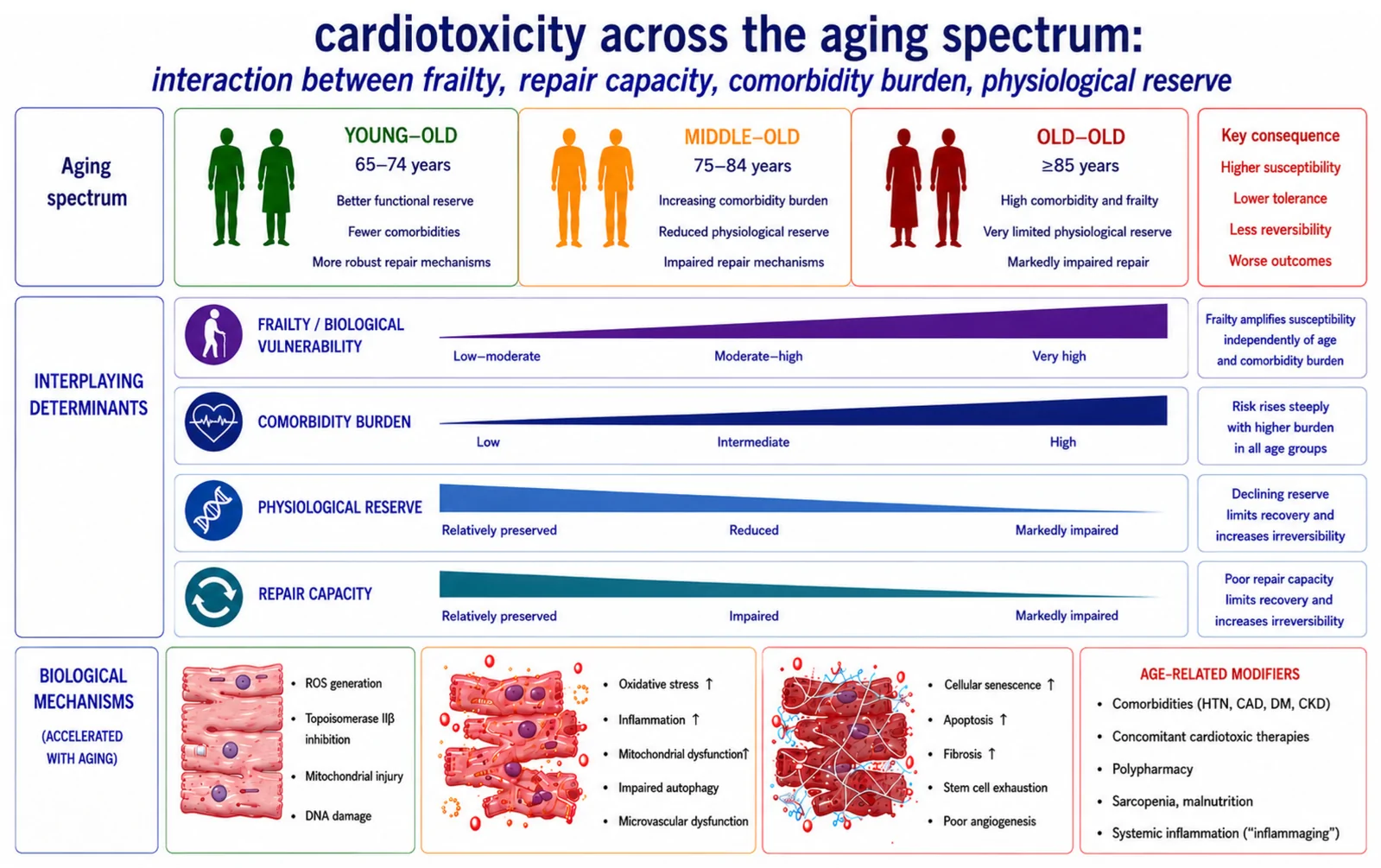
Cardiotoxicity across the aging spectrum: interaction among frailty, repair capacity, comorbidity burden and physiological reserve decline. Abbreviations: CAD, coronary artery disease; CKD, chronic kidney disease; DM, diabetes mellitus; HTN, hypertension; ROS, reactive oxygen species. These factors are interrelated but should not be interpreted as progressing uniformly or in parallel with chronological age.

Aging itself amplifies the risk of CTR-CVT through diminished cardiac reserve, impaired repair mechanisms, chronic inflammation, frailty, multimorbidity, polypharmacy, increased pre-existing CVD, and cumulative exposure to traditional cardiovascular risk factors. Taken together, these biological and clinical factors help explain why older adults are especially susceptible to treatment-related cardiovascular injury and why standard cardio-oncology strategies may not fully address their needs [1,3,6,10] (Figure 1).

### 3.1. Alteration of Repair Mechanisms and Baseline Myocardial Vulnerability

Age-related alterations in cellular repair mechanisms and turnover represent fundamental biological processes underlying the increased vulnerability to cardiotoxicity in elderly cancer patients. DNA repair capacity, mitochondrial function, and proteostasis progressively decline with advancing of years and the natural degenerative process [24,25]. The time-dependent accumulation of cellular damage is widely considered the general cause of aging and may be characterized by genomic instability and telomere attrition, epigenetic alterations, altered intercellular communication, stem cell exhaustion, and dysregulation. Additionally, oxidative stress and chronic low-grade inflammation are increased. The response to myocardial and vascular injury is impaired due to the decreased ability of cardiomyocytes and endothelial cells to adapt to stress and noxious stimuli, such as chemotherapy. Altered cellular turnover and reduced regenerative potential limit myocardial recovery following damage from agents such as anthracyclines and radiation therapy [13,23,24,25,26,27].

Beyond impaired cellular repair pathways, aging is also characterized by progressive cardiovascular remodeling and reduced physiological reserve [28,29,30]. Chronic neurohormonal activation, endothelial dysfunction, increased arterial stiffness, and myocardial fibrosis contribute to age-related changes in cardiac structure and function [28,29,30,31,32]. Importantly, older adults may present subclinical or previously undiagnosed cardiovascular abnormalities, particularly heart failure with preserved ejection fraction (HFpEF), characterized by impaired diastolic relaxation, increased ventricular stiffness, and limited ability to tolerate additional hemodynamic stress despite preserved left ventricular ejection fraction (LVEF) [28,29,30,31,32,33].

This pre-existing myocardial vulnerability may influence the response to antineoplastic therapies. In older adults, CTR-CVT may therefore represent not only a direct consequence of treatment-related myocardial injury but also the interaction between anticancer exposure and an already vulnerable cardiovascular system. Reduced regenerative capacity and impaired adaptation may limit recovery following cardiomyocyte injury, potentially increasing the risk of persistent left ventricular dysfunction, arrhythmias, and functional decline [28,29,30,31,32,33].

### 3.2. Frailty, Multimorbidity, and Biological Vulnerability

Frailty and multimorbidity represent interconnected determinants of vulnerability in older adults with cancer and are particularly relevant in cardio-oncology. Frailty is a multidimensional geriatric syndrome characterized by decreased physiological reserve and increased susceptibility to stressors, including anticancer therapies and cardiovascular insults. It may be conceptualized through different models, including the phenotypic model, which focuses on physical characteristics such as weight loss, exhaustion, weakness, reduced walking speed, and low physical activity, and the deficit accumulation model, which considers the cumulative burden of health deficits over time. Frailty has been consistently associated with increased treatment intolerance, hospitalization, functional decline, and mortality in older adults with cancer [3,10,34,35,36].

Multimorbidity, defined as the coexistence of multiple chronic conditions, frequently accompanies frailty and contributes to the complexity of cardio-oncology decision-making. Older cancer patients commonly present with CVD such as coronary artery disease (CAD), atrial fibrillation, heart failure with preserved or reduced ejection fraction (HFpEF, HFrEF), as well as non-cardiovascular conditions, including chronic kidney disease, diabetes mellitus (DM), chronic obstructive pulmonary disease, and cognitive impairment. The coexistence of these conditions may reduce physiological reserve, modify treatment tolerance, and increase susceptibility to CTR-CVT [3,10,37,38,39].

It is important to note that biological age is a multidimensional concept reflecting an individual’s biological and physiological aging and may differ substantially from chronological age. Although related, biological age should be distinguished from frailty, which reflects increased vulnerability to stressors due to reduced physiological reserve, and from functional status, which refers to an individual’s ability to perform daily activities and maintain independence. CGA is a multidomain clinical assessment that integrates several geriatric domains rather than a measure of biological age itself [10,34,35,36]. The interaction between frailty and multimorbidity creates a complex clinical scenario in which the risk associated with anticancer therapy cannot be predicted by CVD burden alone. Therefore, individualized assessment should incorporate functional status, comorbidity burden, life expectancy, and patient priorities rather than relying solely on chronological age or conventional cardiovascular parameters [34,35,36,37,38,39,40].

### 3.3. Polypharmacy

Polypharmacy, commonly defined as the concurrent use of five or more medications, is highly prevalent among older adults with cancer and represents a significant contributor to vulnerability in cardio-oncology [41,42]. Aging is associated with pharmacodynamic and pharmacokinetic changes, including reduced renal and hepatic clearance, altered drug distribution, increased receptor sensitivity which heightens the risk of drug-to-drug interactions, and adverse pharmacological reactions. In this context, polypharmacy increases clinical complexity by creating potential drug–drug interactions, treatment intolerance, and challenges in medication adherence. Moreover, inappropriate prescribing and the use of potentially inadequate medications, as highlighted by tools such as the Beers Criteria, further compound these risks. Studies have demonstrated that polypharmacy is independently associated with increased hospitalization, treatment intolerance, and mortality in older cancer patients. It is important to note that appropriate cardiovascular therapies remain essential for the optimization of baseline cardiovascular status and prevention of CTR-CVT. The challenge in older adults is to balance evidence-based cardioprotective strategies with altered pharmacokinetics, renal function, hypotension risk, and competing treatment priorities, and that necessitates careful medication reconciliation and individualized risk assessment [33,40,41,42,43,44].

### 3.4. Lifetime Exposure to Cardiovascular Risk Factors

As life expectancy increases, older adults are subjected to prolonged cumulative exposure to traditional cardiovascular risk factors, including arterial hypertension (HTN), diabetes mellitus, dyslipidemia, and tobacco use. However, CVD and cancer share many overlapping risk factors. Chronic exposure to these risk factors and their burden contributes to a heightened baseline cardiovascular risk and predisposes older adults to both overt and subclinical CVD. In the context of cardio-oncology, such cumulative exposure may amplify susceptibility to treatment-related cardiotoxicity, thereby complicating risk stratification and therapeutic decision-making [1,24].

## 4. Critical Appraisal of the Evidence Base

Despite advances in cardio-oncology, the current evidence base remains limited in its applicability to older adults. Most available studies rely on age cutoffs such as 65 years, which mask clinically important differences among patients of different age groups (younger-old, middle-old, and oldest-old). As a result, many conclusions are still extrapolated from younger populations, reducing the applicability of trial findings to real-world geriatric practice [1,3,8,9,45]. This underrepresentation is typically caused by organ function thresholds, performance status, and protocol complexity, rather than chronological age-based exclusion criteria [8,9].

Consequently, trial populations often reflect biologically young and more robust individuals rather than old and frail patients. This discrepancy raises significant questions about the external validity and the generalizability of the study results, especially for those focused on cardiovascular toxicity outcomes, where the baseline cardiovascular assessment has a significant impact [7,9,10]. It is important to note that cardiotoxicity may be presented in older adults atypically, may be underdiagnosed, or interact synergistically with pre-existing CVD and other morbidities, or may aggravate frailty and cachexia, complicating the risk assessment and the treatment protocol and options [1,3,7,8,9,10].

Anthracycline cardiotoxicity in the older adults is clearly age-associated, but current evidence is disproportionately derived from young–old populations (65–74 years), with progressively diminishing data based on age stratification. This creates a knowledge gradient, in which patients at highest risk and greatest vulnerability (≥75–85 years old) are paradoxically those least studied and excluded from the majority of the trials and clinical studies. This highlights the need for frailty-integrated, age-stratified prospective studies. Although multiple large datasets confirm increased cardiotoxicity risk in older adults, none provide granular stratification across the elderly subgroups. Absence of frailty integration, limited representation of patients ≥75 years, and near-complete exclusion of those ≥85 years old represent critical barriers to risk-adapted cardio-oncology care [46,47,48,49,50,51,52,53,54] (Table 1).

As in anthracyclines, across immune checkpoint inhibitors and HER2-targeted therapies, cardiotoxicity is increasingly recognized as a clinically relevant adverse effect. While HER2inhibitor-related cardiotoxicity is predominantly characterized by reversible left ventricular dysfunction and is better described in real-world elderly cohorts, ICI-associated toxicity is rare but often severe, with myocarditis carrying high mortality. Furthermore, clinical trials evaluating ICIs and HER2-targeted therapies have similarly failed to adequately incorporate frailty assessment or biological age metrics, while patients aged ≥85 years remain almost entirely underrepresented. Consequently, the translational gap between geriatric cardio-oncology practice and the evidence generated by oncology clinical trials persists across these therapeutic classes [17,55,56,57,58,59,60,61,62]. (Table 2, Table 3 and Table 4).

**Table 1:** Key clinical studies evaluating anthracycline-induced cardiotoxicity in adult and older adult populations.

Study	Population (Patients)	Age Focus	Design	Anthracycline Exposure	Main Cardiovascular Findings	Older Adult Relevance
Swain et al., 2003 [46]	630	All ages; 27% >65 years	Retrospective analysis of three prospective Phase III trials	Doxorubicin (dose-dependent)	HF incidence rises with cumulative dose; up to ~26% at high doses,	Age important risk factor for CHF, limited characterization of the >85 years
Pinder et al., 2007 [48]	43,338	≥66 (Medicare cohort)	Population-based (SEER–Medicare)	Adjuvant anthracyclines	Increased CHF risk vs. non-anthracycline regimens	First large real-world elderly cohort; no separate analyses, patients >80 years were excluded
Hershman et al., 2008 [47]	9438 (3164 doxorubicin-based chemotherapy)	≥65 years	Retrospective cohort study	Anthracyclines ± others	Higher HF risk in older patients, especially with comorbidities	Direct evidence in patients ≥65 years; advancing age and CV comorbidities independently increased CHF risk
Chavez-MacGregor et al., 2013 [61]	9537 (5229 Anthracycline-based chemotherapy)	≥66 years	Population-based retrospective cohort (SEER–Medicare and Texas Cancer Registry–Medicare)	Adjuvant chemotherapy ± trastuzumab	Anthracycline therapy increased CHF risk; Advanced age is associated with higher cardiotoxicity risk	Large elderly-specific real-world cohort; provides specific evidence of increased CHF risk in patients >80 years
Cardinale et al., 2015 [50]	2625	Mixed-age population	Prospective cohort study	Anthracyclines	Early detection and prompt therapy of cardiotoxicity was associated with substantial LVEF recovery	No age-stratified subgroup analysis
Thavendiranathan et al., 2014 [51]	1504 (384 Anthracycline-based chemotherapy)	Mixed-age popu-lation	Systematic review	Anthracyclines ± others	GLS detects subclinical dysfunction earlier than LVEF, a 10–15% decline in GLS predicted subsequent cardiotoxicity	No elderly-specific analysis; limited age-specific applicability
Zamorano et al., 2016 [27]	Expert consensus	All ages	Position paper	Anthracyclines ± others	Recognizes age as an important risk modifier	No age-specific recommendations, recognized increased risk in older adults
Lyon et al., 2022 [1]	Guideline	All ages	Guideline	Anthracyclines ± others	Emphasizes baseline CV risk assessment and monitoring	Provides limited age-specific evidence and no dedicated geriatric/frailty-based management

Abbreviations: CHF, congestive heart failure; CV, cardiovascular; GLS, global longitudinal strain; HF, heart failure; LV, left ventricular; LVEF, left ventricular ejection fraction; NR, not reported; SEER, Surveillance, Epidemiology, and End Results Program.

**Table 2:** Key clinical studies evaluating cardiotoxicity of immune checkpoints inhibitors (ICIs) in adult and older adult populations.

Study	Therapy Class	Population	Age Focus	Design	Main Cardiovascular Toxicity Findings	Older Adult Relevance
Mahmood et al., 2018 [17]	ICIs	35 ICI-myocarditis cases/105 ICI-treated controls	Mixed-age popu-lation	Multicenter observational registry	ICI myocarditis prevalence ~1.14%; MACE in 46% of myocarditis cases	No elderly-specific or age-stratified analysis
Salem et al., 2018 [55]	ICIs	VigiBase reports, WHO database (31,321 ICSRs ICIs associated)	Broad	Pharmacovigilance, observational, retrospective study	Myocarditis, pericarditis, arrhythmias, vasculitis and HF reported	No elderly-specific or age-stratified risk analysis
Lyon et al., 2018 [56]	ICIs	N/A (Review)	All ages	Expert review	Defines spectrum of immune-mediated CV toxicities (myocarditis, pericarditis, HF, HTN, TE)	No elderly-specific analysis; limited age-specific evidence.
Dolladille et al., 2020 [57]	ICIs	24,079 ICI-associated irAE cases; 6123 rechallenges	Broad	Pharmacovigilance cohort/VigiBase study	Recurrence after ICI rechallenge (~29%)	No elderly-specific or age-stratified rechallenge analysis

Abbreviations: CV, cardiovascular; FAERS, Food and Drug Administration Adverse Event Reporting System; HF, heart failure; HTN, hypertension; ICI, immune checkpoint inhibitor; irAE, immune-related adverse event; ICSRs, individual case safety reports; MACE: major adverse cardiovascular events; N/A, non-available; TE, thromboembolic events; VigiBase, World Health Organization global pharmacovigilance database.

**Table 3:** Key clinical studies evaluating cardiotoxicity of HER2-targeted therapies in adult and older adult populations.

Study	Therapy Class	Population	Age Focus	Design	Main Cardiovascular Toxicity Findings	Older Adult Relevance
Slamon et al., 2001 [58]	HER2 inhibitors (trastuzumab)	469 patients with metastatic breast cancer	Mixed adult population	Randomized controlled trial	LV dysfunction highest with trastuzumab + anthracycline/cyclophosphamide	Older patients underrepresented
Seidman et al., 2002 [59]	HER2 inhibitors (trastuzumab)	Clinical trial cohort	Mixed adult population	Retrospective pooled analysis	Higher HF risk with anthracycline plus trastuzumab	No dedicated elderly subgroup or age-stratified cardiotoxicity analysis
Bowles et al., 2012 [60]	HER2 inhibitors (trastuzumab)	12,500 patients (554 received trastuzumab)	Broad	Population-based retrospective cohort	HF/CM risk increased with anthracyclines and trastuzumab; cumulative HF/CM incidence increased with age	Strongest elderly real-world dataset
Chavez-MacGregor et al., 2013 [61]	HER2 inhibitors (trastuzumab)	9537 patients (2203 received trastuzumab)	≥66 years	Population-based retrospective cohort (SEER–Medicare and Texas Cancer Registry)	Advanced age is associated with higher cardiotoxicity risk	Large elderly-specific real-world cohort
Chen et al., 2012 [62]	HER2 inhibitors (trastuzumab)	45,537 patients with early breast cancer (868 Trastuzumab)	67–94 years	Population-based retrospective cohort (SEER–Medicare)	Cumulative HF risk increases with age and anthracycline exposure	Direct elderly-specific evidence, including women ≥80 years;

Abbreviations: CM: cardiomyopathy; HER2, human epidermal growth factor receptor 2; HF, heart failure; LV, Left Ventricular; RCT, randomized controlled trial; SEER, Surveillance, Epidemiology, and End Results.

**Table 4:** Methodological limitations and evidence gaps across major cardio-oncology therapies in older adults.

Domain	Anthracyclines	HER2-Targeted Therapies	Immune Checkpoint Inhibitors (ICIs)	Clinical Implications
Age stratification	Limited and heterogeneous; mainly ≥65 cut-offs	Age-specific analyses in selected observational cohorts	Rare age-specific stratification	No consistent stratification across gerontological age groups (65–74, 75–84, ≥85 years)
≥75 years representation	Limited	Underrepresented	Underrepresented	75–84 years populations remain insufficiently characterized
≥85 years representation	Almost absent (Very limited; no dedicated ≥85-year analyses)	Virtually absent (Present in some observational cohorts, but rarely analyzed separately)	Minimal representation (No dedicated ≥85-year subgroup analyses)	No evidence base for ≥85 years patients
Study design and selection	Predominantly observational retrospective or registry-based	Mixed RCTs and observational cohorts	Registry/pharmacovigilance studies	Selection bias and healthy-user effect frequently present
Cardiac phenotyping	LVEF-based monitoring dominant	Predominantly LVEF-based assessment and clinical HF/CM endpoints	Heterogeneous use of imaging, ECG, biomarkers	Subclinical dysfunction may remain undetected
Biomarkers	Limited validation in older adult	Limited validation in older trastuzumab-treated patients	Age-specific biomarker thresholds not standardized	Limited age-specific surveillance strategies
Frailty integration	Rarely incorporated	Rarely incorporated	Rarely incorporated	Frailty-informed risk assessment remains limited
Endpoints assessed	HF/LVEF decline (primary outcomes)	HF/LVEF decline (primary endpoint)	Myocarditis-related outcomes, arrhythmias, vasculitis, MACE	Quality of life, functional decline and geriatric outcomes underreported
Follow-up duration	Heterogeneous; long-term follow-up only in selected observational cohorts	Variable; multi-year follow-up available in selected observational cohorts	Variable, often insufficient; evidence focused predominantly on early events	Late-onset cardiotoxicity likely underestimated, especially ≥75–85 years
Treatment heterogeneity	Variable cumulative doses and regimens	Variable trastuzumab exposure, duration, sequencing, and concomitant therapies	Diverse agents and combination strategies	Limits comparability across studies and generalizability
Clinical trials involvement	Younger populations predominantly	Elderly frequently underrepresented, elderly-specific evidence mainly observational	Elderly frequently underrepresented	Persistent evidence gap in ≥75 and ≥85 years age groups

Abbreviations: WHO: World Health Organization; RCT: randomized controlled trial; LVEF: left ventricular ejection fraction; HF: heart failure; ECG: electrocardiogram; BNP: B-type natriuretic peptide; ICIs: immune checkpoint inhibitors; **CM: cardiomyopathy; MACE: major adverse cardiovascular even**.

Beyond anthracyclines, HER2-targeted therapies, and ICIs, several additional anticancer therapies have emerged as important contributors to cardiovascular toxicity in older adults. Vascular endothelial growth factor (VEGF) inhibitors and tyrosine kinase inhibitors have been associated with HTN, heart failure, thromboembolic events, and arrhythmias, whereas proteasome inhibitors, Bruton tyrosine kinase inhibitors, androgen deprivation therapy, CAR-T therapies, and radiotherapy have also been linked to clinically relevant cardiovascular complications [1,2,3,63,64,65,66]. However, evidence regarding these treatment classes in older adult populations remains limited and is predominantly derived from retrospective studies, heterogeneous cohorts, or extrapolation from younger and biologically fitter populations. Moreover, age-stratified analyses, frailty assessments, geriatric-specific outcomes, and prospective data in very old individuals are largely unavailable, representing additional unmet needs in geriatric cardio-oncology research [1,2,3,8,63,64,65,66].

According to the current cardio-oncology guidelines, international expert consensus, and specific scientific statements older adults are recognized as a particularly vulnerable population with increased susceptibility to treatment-related cardiovascular complications. Current recommendations emphasize individualized cardiovascular assessment, taking into account frailty, comorbidity burden, functional status, and patient preferences [1,2,8,64,65]. Nevertheless, validated geriatric-specific thresholds, surveillance strategies, and management algorithms remain limited, particularly for frail individuals and oldest-old populations. It is important to notice that these limitations primarily reflect the paucity of geriatric-specific evidence rather than the shortcomings of current guideline recommendations [1,2,3,7].

### Gaps in Evidence: Beyond Cardiovascular Endpoints Toward Patient-Centered Outcomes

The existing gaps and limitations of the current cardio-oncology evidence base are conceptual rather than just quantitative. Beyond the very limited representation of elderly patients and the frequent exclusion of the populations >85 years from clinical trials, several additional limitations hinder the interpretation and applicability of the available evidence. As highlighted in Table 4, the available literature frequently relies on broad age cutoffs rather than stratification into clinically meaningful older-age subgroups, thereby obscuring substantial heterogeneity in biological aging, frailty burden, functional status, and treatment tolerance [3,8,9,10].

The current cardio-oncology literature relies on trial populations and cardiotoxicity endpoints that are not originally designed for geriatric oncology. Oncologic studies predominantly focus on cancer-related endpoints, including overall survival, progression-free survival, treatment completion, and treatment interruption. Cardiology studies frequently emphasize cardiovascular events and imaging parameters, while geriatric medicine focuses on frailty trajectories, functional independence, cognition, hospitalization, and quality of life. The lack of integration among these domains may oversimplify complex treatment decisions in elderly cardio-oncology patients. Across therapeutic classes, the evidence is methodologically heterogeneous and includes retrospective cohorts, post hoc analyses, pharmacovigilance studies, prospective observational studies, and selected clinical trial populations, with variable sample sizes and follow-up durations [17,46,47,48,49,50,51,52,53,54,55,56,57,58,59,60,61,62]. Differences in cancer type, treatment exposure, definitions of cardiotoxicity, surveillance protocols, baseline cardiovascular risk, and comorbidity burden further complicate comparisons and limit generalizability [3,7,8,9,10]. Older adults frequently experience competing risks that alter both absolute risk estimates and clinical relevance of endpoints [3,40].

Important gaps also remain regarding treatment characterization and outcome assessment. Standardization of cumulative therapeutic dose exposure, cardioprotective interventions, surveillance protocols, and comparator groups is frequently lacking, while randomized controlled trials specifically designed for older adult populations remain scarce [1,3,8,9,10,46,47,48,49,50,51,52,53,54,55,56,57,58,59,60,61,62]. In addition, available studies predominantly focus on conventional cardiovascular outcomes, including left ventricular dysfunction, biomarker abnormalities, heart failure, and cardiovascular mortality, whereas late cardiovascular events, treatment completion, functional decline, frailty trajectories, quality of life, cognitive outcomes, and other geriatric-specific endpoints are seldom systematically evaluated. Consequently, the current evidence may underestimate the broader clinical impact of CTR-CVT in older adults and fails to fully capture outcomes that are particularly relevant for patient-centered decision-making in geriatric cardio-oncology [3,8,9,10,40,64,65].

## 5. Methodological and Conceptual Limitations

The evidence gaps identified above extend beyond the underrepresentation of older adult patients and insufficient age stratification. They also reflect important methodological and conceptual challenges that limit the applicability of current cardio-oncology approaches to older adults. Many diagnostic tools, surveillance strategies, and risk prediction models currently used in cardio-oncology have been developed and validated in younger or biologically fitter populations, potentially reducing their accuracy and clinical relevance in frail older adults [1,2,3,10].

### 5.1. Diagnostic Challenges: Biomarkers and Imaging in Frail Older Adult Patients

The diagnosis and monitoring of CTR-CVT in older adults present unique challenges due to age-related physiological changes, comorbid conditions, and frailty. In older adult, the interpretation of both biomarkers and cardiac imaging requires careful contextualization, as conventional thresholds may be less specific and less predictive of clinically meaningful outcomes [1,3,10].

Cardiac biomarkers, such as high-sensitivity troponin and natriuretic peptides (BNP/NT-proBNP), are widely used for the early detection of subclinical myocardial injury in cardio-oncology. In frail elderly patients, elevated baseline values of cardiac biomarkers are common, due to chronic structural heart disease, renal dysfunction, or systemic inflammation, reducing diagnostic specificity [1,3,10,66,67]. Age-related reductions in renal clearance and the increased prevalence of heart failure (HFrEF, HFpEF) may interfere with the interpretation of biomarker changes, making relative trends more informative and less diagnostic for cardiotoxic effects of antineoplastic agents [3,10,66]. Elevated baseline troponin and natriuretic peptide concentrations in older adults should be interpreted cautiously, as they may reflect underlying renal dysfunction, HFpEF, atrial fibrillation, systemic inflammation, frailty, or cancer-related factors rather than treatment-induced cardiovascular toxicity alone. Consequently, serial measurements and longitudinal trends may provide greater clinical utility than isolated absolute values, particularly in older adults with multimorbidity and competing cardiovascular conditions [1,66,67].

Similarly, imaging-based assessment, especially echocardiography with LVEF and global longitudinal strain (GLS), remains central to cardiotoxicity surveillance. Moreover, the limited ability to comply with imaging protocols, or baseline anatomical abnormalities that diminish interpretability may restrict image quality in frail older persons with many comorbidities [1,68]. Although GLS may detect subclinical myocardial dysfunction earlier than conventional echocardiographic parameters, surveillance strategies in older adults should be individualized according to baseline cardiovascular risk, frailty burden, life expectancy, and treatment intent. Therefore, surveillance intensity should be guided by potential clinical utility rather than chronological age or frailty status alone [1,3,68].

Furthermore, due to logistical limitations, claustrophobia, renal dysfunction, the presence of metallic prostheses, implanted cardiac devices, or low clinical feasibility in frail individuals, advanced imaging modalities like cardiac magnetic resonance imaging (CMR) may be underutilized in older adult patients. This creates a diagnostic gap in precisely characterizing myocardial injury in the population at the greatest risk.

All of these limitations point to the necessity of age-appropriate diagnostic techniques that combine imaging and biomarkers with CGA and clinical context. A dynamic, longitudinal, and customized interpretive approach may enhance clinical decision-making and diagnostic accuracy in frail older patients rather than depending only on absolute biomarker thresholds or imaging cutoffs [1,3,10].

### 5.2. Management Strategies and Multidisciplinary Models

Optimal management of cardiovascular risk in older adults undergoing cancer therapy requires a multidisciplinary, patient-centered approach integrating oncology, cardiology, and geriatric medicine. The complexity of this population stems from the coexistence of multimorbidity, frailty, polypharmacy, and variable life expectancy, necessitating individualized treatment strategies rather than protocol-driven care [1,2,3,10,40].

A cornerstone of contemporary cardio-oncology practice is the establishment of dedicated multidisciplinary cardio-oncology teams, which facilitate early cardiovascular risk stratification, baseline optimization, and longitudinal monitoring throughout cancer treatment. Evidence suggests that such integrated care models improve early detection of cardiotoxicity and may reduce treatment interruptions, although robust randomized data remain limited, particularly in older adults [1,2,7]. In frail older adults, the inclusion of geriatric assessment within these teams further enhances clinical decision-making by incorporating functional status, cognition, and patient priorities into therapeutic planning [3,10].

**Figure 2:**
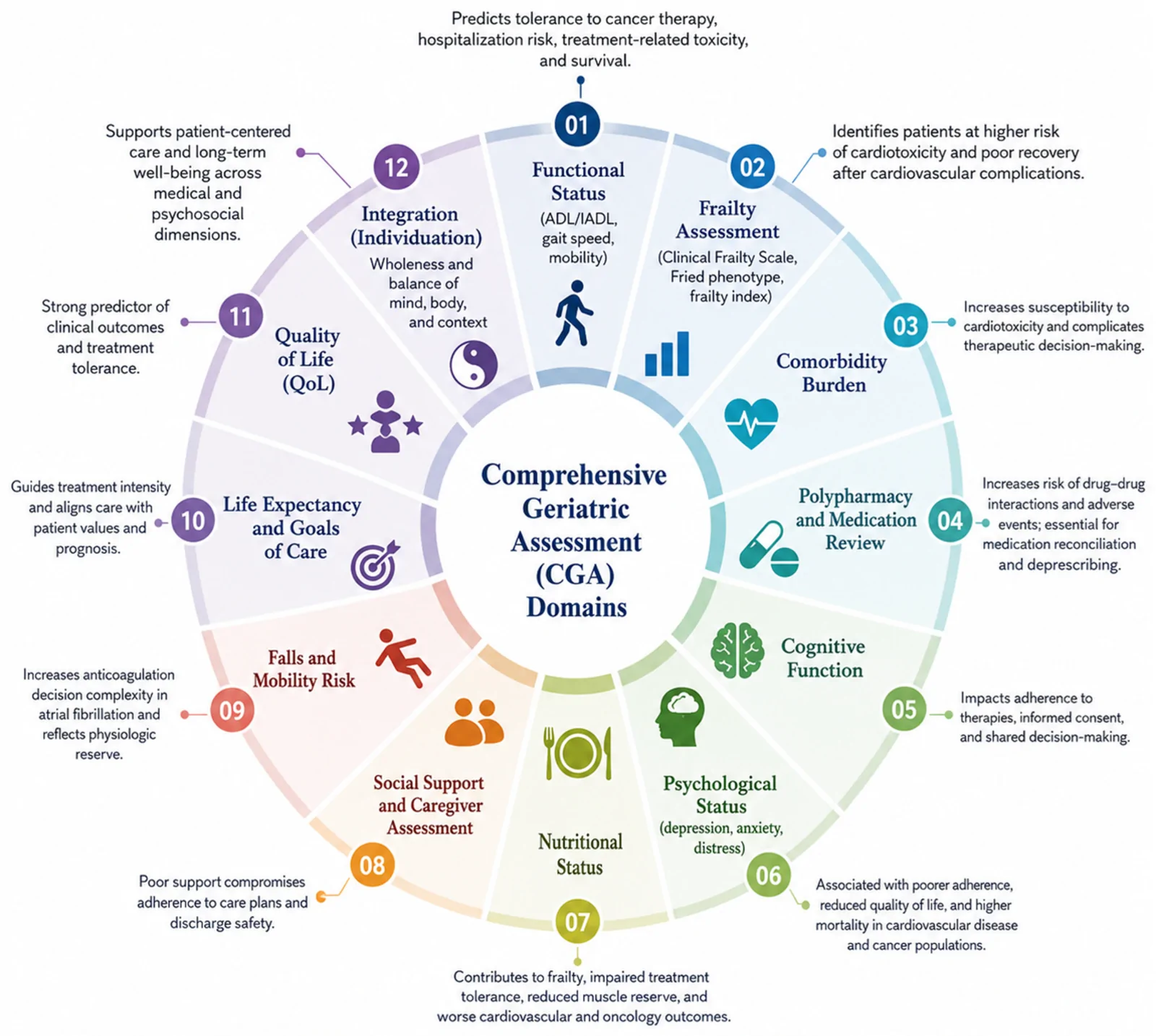
CGA integration in cardio-oncology: A multidimensional evaluation that may inform risk stratification and supports individualized, patient-centered clinical decision-making in older adults with cancer.

The CGA is a complex, multidisciplinary assessment used to assess functional status in older adults, frailty, comorbidities, cognition, nutritional status, psychological health, social support, and polypharmacy. Beyond chronological age, CGA offers clinically significant information in cardio-oncology, allowing for the personalized assessment of treatment tolerance, cardiovascular vulnerability, and competing mortality risks. Increased chemotherapy toxicity, hospitalization, cardiovascular problems, and death in older cancer populations have been independently linked to a number of CGA categories, including frailty, functional impairment, and multimorbidity [1,2,3,10,40,69]. Incorporating CGA into cardio-oncology practice may support shared decision-making and facilitate individualized, patient-centered therapeutic approaches in older adults receiving cancer treatment (Figure 2).

From a practical perspective, the integration of CGA into cardio-oncology practice may be facilitated by the use of brief and easily applicable screening tools. The G8 screening tool is widely adopted in geriatric oncology to identify older patients who may benefit from a more comprehensive assessment, whereas the Clinical Frailty Scale (CFS) offers a rapid evaluation of frailty based on functional status and degree of dependence. Physical frailty can also be characterized using the Fried frailty phenotype, which encompasses weight loss, exhaustion, weakness, reduced gait speed, and low levels of physical activity. In addition, the Cancer and Aging Research Group (CARG) toxicity score may assist in estimating the risk of treatment-related toxicity, while the Charlson Comorbidity Index (CCI) provides a structured assessment of comorbidity burden and competing health risks. Collectively, these tools may represent a feasible minimum geriatric assessment framework for cardio-oncology clinics, helping clinicians incorporate frailty, comorbidities, treatment tolerance, and patient priorities into individualized cardiovascular and oncologic decision-making [34,70,71,72,73,74].

### 5.3. Cardiotoxicity Surveillance and Cardioprotection in Older Adult Patients: Towards Individualized, Risk-Adapted Care

Current approaches to cardiotoxicity surveillance are largely based on treatment-specific risk categories and standardized monitoring protocols. However, their application in older adults requires further individualization. Elderly patients represent a heterogeneous population in which the potential benefit of early cardiotoxicity detection must be balanced against the burden of repeated assessments, competing comorbidities, life expectancy, and overall treatment goals [1,3,10,69].

In older adults, risk-adapted management should integrate baseline cardiovascular risk, cancer prognosis, frailty status, and patient preferences. Pharmacologic treatment remains a key component of primary prevention of CTR-CVT, the early intervention and the management of the established CVD, although its efficacy in elderly populations is less well established. Primary prevention of CTR-CVT should focus on optimization of baseline cardiovascular risk factors and adherence to current cardio-oncology guideline recommendations [1]. Dexrazoxane remains the only pharmacological agent with regulatory approval for the prevention of anthracycline-induced cardiotoxicity in selected patients receiving high cumulative anthracycline doses [1]. Non-pharmacologic strategies, including structured physical activity, optimization of cardiovascular risk factors, such as blood pressure and glycemic control, and nutritional support, also play an important role in reducing cardiovascular vulnerability and maintaining functional independence. Exercise-based interventions have demonstrated benefits in improving functional capacity and quality of life in older cancer patients, although implementation remains inconsistent in clinical practice [1,75].

Early detection and timely intervention are key components of effective management of CTR-CVT. Angiotensin-converting enzyme inhibitors (ACE inhibitors), angiotensin receptor blockers (ARBs), and beta-blockers have demonstrated benefits and may attenuate cardiac dysfunction in selected patients receiving anthracyclines or HER2-targeted therapies, particularly when initiated early or guided by biomarker or imaging changes [1,69,76,77,78]. Nevertheless, the implementation of cardioprotective strategies in older adults remains challenging due to the heterogeneity of this population and the frequent coexistence of baseline hypotension, blood pressure variability, renal dysfunction, multimorbidity, and polypharmacy.

Importantly, cardioprotection in older adults should not be viewed solely as the addition of further pharmacological agents, but rather as a process of individualized cardiovascular optimization. Decisions regarding ACE inhibitors, ARBs, beta-blockers, statins, dexrazoxane, or treatment modification strategies should take into account baseline cardiovascular risk, frailty burden, competing comorbidities, treatment goals, life expectancy, and patient preferences. In this setting, the potential benefits of cardioprotective interventions must be carefully weighed against the risks of hypotension, renal dysfunction, falls, polypharmacy, and reduced treatment tolerance. In selected high-risk individuals, dose adjustment, treatment de-escalation, the use of less cardiotoxic regimens, or dexrazoxane administration may represent reasonable strategies to mitigate cardiovascular toxicity while preserving oncologic efficacy [1,2,3,49,69,79,80,81,82].

Similarly, statins have emerged as potential cardioprotective agents due to their pleiotropic anti-inflammatory and endothelial stabilizing effects. Observational data suggest a reduction in cardiovascular-toxicity risk among statin users, although randomized evidence remains limited and age-specific outcomes are insufficiently characterized [69,80,82,83]. Importantly, the risk–benefit balance of cardioprotective pharmacotherapy in older adults must consider competing risks, treatment tolerance, and overall goals of care. Treatment modifications, including dose adjustment, schedule alteration, or selection of less cardiotoxic regimens, may be appropriate in selected high-risk individuals, particularly when curative benefit is marginal or life expectancy is limited [2,3].

It is important to clarify that management of the established CVD should follow contemporary cardiovascular guidelines while taking into account frailty, comorbidities, treatment goals, and potential drug interactions [1].

### 5.4. Long-Term Survivorship, Cardiovascular Aging and Palliative Care

The number of elderly cancer survivors is increasing dramatically as advances in cancer therapy continue to improve survival, raising attention to the long-term cardiovascular consequences resulting from cancer treatment. In cardio-oncology, survivorship extends beyond acute phases of cardiotoxicity and includes long-term cardiovascular complications that may appear years or decades after treatment [1,84,85,86]. In older adults, radiation-associated CVD is a significant survivorship concern. Progressive endothelial dysfunction, arterial stiffness, accelerated atherosclerosis, valvular degeneration, microvascular ischemia, and pericardial fibrosis are all potential side effects of irradiation that frequently develop years after exposure [23,87]. Chronic cumulative exposure to anthracyclines may also be linked to progressive heart failure, decreased diastolic function, and late myocardial fibrosis [1,80]. Crucially, survivorship-related cardiovascular impairment in older adults may manifest in the form of progressive frailty, decreased exercise tolerance, sarcopenia, and loss of functional independence in addition to overt CVD [31,84,86,88]. These outcomes may clinically be more relevant in geriatric populations than isolated imaging abnormalities or biomarker elevations [84,86,89].

In order to maintain functional capacity and quality of life, survivorship care for elderly cardio-oncology patients should include long-term cardiovascular surveillance, optimization of modifiable cardiovascular risk factors, structured exercise interventions, and thorough geriatric assessment. However, aggressive oncologic or cardiologic interventions may only slightly improve survival in frail older adults with advanced cancer, severe cardiovascular disease, or other comorbidities, while significantly raising symptom burden, hospitalization risk, and functional decline. As a result, in addition to conventional oncologic and cardiovascular objectives, treatment choices should take into account patient-centered outcomes like quality-adjusted survival, symptom control, preservation of independence, and cognitive function [1,84,86,89,90].

CGA may facilitate these discussions by identifying frailty, cognitive vulnerability, nutritional deficits, and social limitations that influence treatment tolerance and prognosis [70,89]. Importantly, palliative care should not be viewed exclusively as end-of-life management, but rather as an integrated supportive approach throughout the disease trajectory [90]. Multidisciplinary collaboration among oncologists, cardiologists, geriatricians, palliative care specialists, patients, and caregivers is therefore essential to achieve individualized and ethically balanced treatment strategies in this highly vulnerable population [1,84,89,90].

## 6. Proposed Geriatric-Integrated Framework for Cardiovascular Assessment and Management in Older Adults Receiving Cancer Therapy

Taken together, these observations underscore the need for a geriatric-integrated approach to cardio-oncology. Current recommendations provide limited guidance regarding age-specific surveillance strategies, treatment adaptation, and patient-centered decision-making in older adults. Indeed, evidence-based data specifically informing the management of older adults with cancer remain scarce. Commonly used gerontological age strata may provide a practical means of describing older populations, while CGA provides a multidimensional assessment of their functional and biological vulnerability. Chronological age should not be considered a rigid determinant of therapeutic decisions or medical management, but rather as a descriptive parameter that may help contextualize physiological reserve, vulnerability, and competing risks. Although it cannot dictate management, age-related context may provide valuable insights into the clinical challenges that patients are likely to encounter across different age and frailty categories and help identify domains requiring closer attention.

In this context, individualized assessment based on biological age, frailty, functional status, and patient preferences may represent the most appropriate strategy for optimizing cardiovascular care in older adults receiving cancer therapy. Therefore, we propose a practical age- and frailty-stratified framework incorporating cardiovascular assessment, geriatric evaluation, surveillance strategies, and treatment considerations to facilitate individualized management in older adults undergoing cancer therapy that can help in understanding and redirecting the needs of a holistic cardio-oncology assessment and follow-up. The proposed framework is not evidence-based, but uses age strata as a pragmatic organizational structure to reflect the heterogeneity of older adults and facilitate discussion of the available evidence. The age groups are used primarily to organize the available evidence rather than to guide treatment decisions. The framework integrates guideline-supported cardio-oncology recommendations with evidence extrapolated from general cardio-oncology populations and expert-informed geriatric considerations, given the limited evidence specifically available in older and frail adults. However, clinical decisions should primarily be guided by biological age, frailty, functional status, cancer prognosis, treatment intent, baseline cardiovascular risk, and patient preferences rather than chronological age alone. We acknowledge that robust evidence is lacking, and, therefore, the framework is intended to support individualized clinical judgment rather than dictate management decisions (Table 5).

Above is our proposed age- and frailty-stratified cardio-oncology management framework in older adults. This framework represents an expert-opinion-informed and pragmatic approach derived from available evidence and geriatric principles, and has not been prospectively validated. The proposed age strata are intended as a pragmatic descriptive framework and should not be interpreted as the primary determinant of management. Clinical decisions should be individualized according to frailty, functional status, comorbidity burden, baseline cardiovascular risk, cancer prognosis, treatment intent, life expectancy, and patient preferences.

## 7. Future Directions

Future research should focus on prospective studies that specifically include older adults, particularly those aged 75 years and older and those with frailty or multimorbidity. CGA should be incorporated into trial design and clinical risk stratification, while future work should also prioritize patient-centered outcomes such as functional preservation, symptom burden, and quality of life, alongside multidisciplinary and risk-adapted care models.

Ultimately, the management of elderly cardio-oncology patients requires a transition from age-based approaches toward biologically and functionally informed care. Expanding real-world registries, prospective geriatric-specific trials, and collaborative international research initiatives will be essential to bridge current evidence gaps and improve equity in cardio-oncology practice. A more individualized and holistic approach may not only improve cardiovascular and oncologic outcomes, but also preserve functional capacity and quality of life in the rapidly growing population of older cancer survivors. The proposed framework should be considered a conceptual and clinically pragmatic tool intended to guide individualized assessment and stimulate future research, rather than a validated management algorithm.

## 8. Conclusions

In conclusion, older adult cancer patients represent a growing and clinically complex population characterized by substantial heterogeneity and vulnerability. The underrepresentation of older adults in cardio-oncology creates important gaps in evidence, and consequently in the baseline assessment, the risk stratification, and management of these patients. Much of the available knowledge continues to be extrapolated from younger or biologically fitter populations, while frailty, multimorbidity, functional reserve, and competing health risks remain inadequately represented in both clinical trials and risk prediction models.

This review highlights that the major challenge in geriatric cardio-oncology extends beyond the prevention of cardiotoxicity alone. A more individualized approach integrating CGA, multidisciplinary care, and patient-centered decision-making is needed to improve both cardiovascular safety and overall outcomes in this growing patient group. Although this framework is not prospectively validated, it may serve as a practical aid for integrating cardiovascular, oncologic, and geriatric considerations into individualized decision-making processes.

Limitations: The present review has several methodological limitations. As a narrative review based on a non-systematic literature search, it may be subject to study-selection bias, and relevant studies may not have been identified or included. In addition, no formal risk-of-bias or quality assessment of the included studies was performed. The heterogeneity of the available evidence, particularly regarding age stratification and the assessment of frailty and functional status, limits direct comparisons across studies. Therefore, the findings and the proposed framework should be interpreted in the context of these limitations.

## Figures and Tables

**Table 5:** Proposed geriatric-integrated cardio-oncology management framework for older adults receiving cancer therapy.

Patient Profile	Baseline Cardiovascular Assessment	Geriatric Assessment (CGA Domains)	Monitoring Strategy	Treatment Considerations
**65–74 years, fit older adult** Fit, independent, limited comorbidity	• Standard CV risk assessment (history, BP, lipids, DM, smoking) • ECG in all patients • Echocardiography with GLS (clinically relevant) • Optimize modifiable CV risk factors	• Frailty screening (e.g., G8, FRAIL) • Functional status (ADL/IADL) • Comorbidity assessment (e.g., CCI) • Medication review (polypharmacy) • Cognitive screening as indicated • Nutritional status	• Guideline-recommended CTR-CVT surveillance according to therapy risk • Echocardiography at baseline and at recommended intervals • Biomarkers (troponin, BNP/NT-proBNP) according to risk and therapy	• Usually standard oncologic therapy • Individualized cardiovascular prevention and risk-factor optimization • Shared decision-making
**65–74 years, vulnerable/frail** Pre-frail or frail, increased vulnerability	• Detailed CV evaluation • Echocardiography with GLS (baseline and serial) • Biomarkers (troponin, BNP/NT-proBNP) at baseline • Evaluate HFpEF risk, arrhythmias and renal function	• Comprehensive CGA • Frailty assessment (e.g., Fried, Rockwood) • Cognitive function • Nutritional status • Mobility, gait and fall risk • Polypharmacy and drug interactions • Psychosocial status	• Surveillance tailored to baseline risk and cancer therapy • Echocardiography (including GLS) when indicated • Serial biomarkers (troponin, BNP/NT-proBNP) • Clinical assessment at each cycle	• Discuss risk–benefit of treatment intensity • Consider guideline-directed cardioprotective strategies • Close collaboration with cardio-oncology team • Shared decision-making
**75–84 years** Older adult, higher comorbidity burden	• High suspicion for occult CV disease • Echocardiography with GLS • Biomarkers at baseline and periodically • Evaluate HFpEF, arrhythmias and renal function • Consider stress testing if indicated	• Formal CGA • Frailty and sarcopenia assessment • Cognitive evaluation • Functional status and independence • Comorbidity burden • Polypharmacy and deprescribing opportunities • Social support	• Individualized imaging and biomarker monitoring • Shorter surveillance intervals for high-risk therapies • Monitor for HF, arrhythmias, ischemia and renal dysfunction • Focus on early detection of subclinical changes	• Balance cancer treatment benefit versus toxicity and functional decline • Consider dose modification, alternative regimens or de-escalation • Optimize comorbidities and medications • Patient goals and preferences
**≥85 years**	• Comprehensive baseline CV assessment according to CV and treatment-related risk • Echocardiography • Biomarkers guided by clinical context	• Full CGA with focus on: –Life expectancy and prognosis –Cognitive status –Functional status –Nutritional status -Comorbidities -Polypharmacy -Social support –Patient values and goals of care	• Individualized surveillance according to baseline CV risk, treatment-related risk, and clinical status • Avoid repeated testing without actionable impact • Prioritize quality of life	• Shared decision-making with patient and caregivers • Consider treatment modification, de-escalation or best supportive care • Focus on comfort, function and patient-centered goals • Treatment decisions based on overall clinical status, cancer prognosis, treatment goals, and patient preferences (age alone should not prompt treatment decisions)
**Frail patients, irrespective of chronological age**	• Focus on symptoms and CV reserve • Baseline CV assessment considering frailty, comorbidity burden, and limited physiological reserve • Echocardiography only if it will change management • Biomarkers guided by clinical context • Avoid burdensome investigations	• Full CGA with focus on: -Life expectancy and prognosis –Frailty severity -Cognitive status –Functional dependence –Nutritional status –Caregiver support/social and resources –Patient values and goals of care -Polypharmacy	• Consider individualized surveillance according to clinical vulnerability, treatment-related risk, and feasibility • Symptom-driven and clinically meaningful monitoring • Minimize monitoring burden • Avoid repeated testing without actionable impact • Prioritize quality of life	• Shared decision-making with patient and caregivers • Consider treatment modification, de-escalation or best supportive care • Focus on comfort, functional status and patient-centered goals • Balance expected oncologic benefit against CV risk, competing risks, functional impact, and patient goals • Consider palliative care • Ethical treatment decisions

Abbreviations: ACEi, angiotensin-converting enzyme inhibitors; ADL, Activities of Daily Living; ARBs, Angiotensin II Receptor Blockers; BP, Blood pressure; BNP, B-type natriuretic peptide; CCI, Charlson Comorbidity Index; CGA, Comprehensive Geriatric Assessment; CTR-CVT, cancer therapy-related cardiovascular toxicity; CV, cardiovascular; DM, diabetes mellitus; ECG, electrocardiogram; FRAIL, Fatigue, Resistance, Ambulation, Illness, and Loss of weight; GLS, Global longitudinal strain; IADL, Instrumental Activities of Daily Living; NT-proBNP, N-terminal pro B-type natriuretic peptide; HF, heart failure.

## Data Availability

No new data were created or analyzed in this study. Data sharing is not applicable to this article.

## References

[1] Lyon A.R., López-Fernández T., Couch L.S., Asteggiano R., Aznar M.C., Bergler-Klein J., Boriani G., Cardinale D., Cordoba R., Cosyns B. (2022). 2022 ESC Guidelines on cardio-oncology developed in collaboration with the European Hematology Association (EHA), the European Society for Therapeutic Radiology and Oncology (ESTRO) and the International Cardio-Oncology Society (IC-OS). Eur. Heart J..

[2] Herrmann J., Lenihan D., Armenian S., Barac A., Blaes A., Cardinale D., Carver J., Dent S., Ky B., Lyon A.R. (2022). Defining cardiovascular toxicities of cancer therapies: An International Cardio-Oncology Society (IC-OS) consensus statement. Eur. Heart J..

[3] Topa E., De Rosa E., Cuomo A., Curcio F., Rizza M., Elia F., Flocco V., Attanasio U., Iengo M., Fiore F. (2025). Cardio-Oncology Challenges in Elderly Patients. J. Clin. Med..

[4] Pilleron S., Soto-Perez-de-Celis E., Vignat J., Ferlay J., Soerjomataram I., Bray F., Sarfati D. (2021). Estimated global cancer incidence in the oldest adults in 2018 and projections to 2050. Int. J. Cancer.

[5] Bray F., Laversanne M., Sung H., Ferlay J., Siegel R.L., Soerjomataram I., Jemal A. (2024). Global cancer statistics 2022: GLOBOCAN estimates of incidence and mortality worldwide for 36 cancers in 185 countries. CA Cancer J. Clin..

[6] Reddy P., Shenoy C., Blaes A.H. (2017). Cardio-oncology in the older adult. J. Geriatr. Oncol..

[7] Bisceglia I., Canale M.L., Silvestris N., Gallucci G., Camerini A., Inno A., Camilli M., Turazza F.M., Russo G., Paccone A. (2023). Cancer survivorship at heart: A multidisciplinary cardio-oncology roadmap for healthcare professionals. Front. Cardiovasc. Med..

[8] Sedrak M.S., Freedman R.A., Cohen H.J., Muss H.B., Jatoi A., Klepin H.D., Wildes T.M., Le-Rademacher J.G., Kimmick G.G., Tew W.P. (2021). Older adult participation in cancer clinical trials: A systematic review of barriers and interventions. CA Cancer J. Clin..

[9] Ludmir E.B., Mainwaring W., Lin T.A., Miller A.B., Jethanandani A., Espinoza A.F., Mandel J.J., Lin S.H., Smith B.D., Smith G.L. (2019). Factors Associated with Age Disparities Among Cancer Clinical Trial Participants. JAMA Oncol..

[10] Hanlon E., Diaz A.N.R., Sedrak M.S., Strom J.B., Asnani A. (2024). Cancer therapy-associated cardiotoxicity: A look at frailty. J. Geriatr. Oncol..

[11] Burns S.D., Crimmins E.M., Zhang M., Ailshire J.A. (2025). Psychosocial Well-Being Differences Between the Young Old, Old-Old, and Oldest Old: A Global Comparison. J. Aging Health.

[12] Qiu Y., Jiang P., Huang Y. (2023). Anthracycline-induced cardiotoxicity: Mechanisms, monitoring, and prevention. Front. Cardiovasc. Med..

[13] Cardinale D., Iacopo F., Cipolla C.M. (2020). Cardiotoxicity of Anthracyclines. Front. Cardiovasc. Med..

[14] Li C., Bhatti S.A., Ying J. (2022). Immune Checkpoint Inhibitors-Associated Cardiotoxicity. Cancers.

[15] Wang D., Bauersachs J., Berliner D. (2023). Immune Checkpoint Inhibitor Associated Myocarditis and Cardiomyopathy: A Translational Review. Biology.

[16] Ronen D., Bsoul A., Lotem M., Abedat S., Yarkoni M., Amir O., Asleh R. (2022). Exploring the Mechanisms Underlying the Cardiotoxic Effects of Immune Checkpoint Inhibitor Therapies. Vaccines.

[17] Mahmood S.S., Fradley M.G., Cohen J.V., Nohria A., Reynolds K.L., Heinzerling L.M., Sullivan R.J., Damrongwatanasuk R., Chen C.L., Gupta D. (2018). Myocarditis in Patients Treated with Immune Checkpoint Inhibitors. J. Am. Coll. Cardiol..

[18] Palaskas N., Lopez-Mattei J., Durand J.B., Iliescu C., Deswal A. (2020). Immune Checkpoint Inhibitor Myocarditis: Pathophysiological Characteristics, Diagnosis, and Treatment. J. Am. Heart Assoc..

[19] Copeland-Halperin R.S., Liu J.E., Yu A.F. (2019). Cardiotoxicity of HER2-targeted therapies. Curr. Opin. Cardiol..

[20] Gonciar D., Mocan L., Zlibut A., Mocan T., Agoston-Coldea L. (2021). Cardiotoxicity in HER2-positive breast cancer patients. Heart Fail. Rev..

[21] Sendur M.A., Aksoy S., Altundag K. (2013). Cardiotoxicity of novel HER2-targeted therapies. Curr. Med. Res. Opin..

[22] Lin M., Xiong W., Wang S., Li Y., Hou C., Li C., Li G. (2022). The Research Progress of Trastuzumab-Induced Cardiotoxicity in HER-2-Positive Breast Cancer Treatment. Front. Cardiovasc. Med..

[23] Mitchell J.D., Cehic D.A., Morgia M., Bergom C., Toohey J., Guerrero P.A., Ferencik M., Kikuchi R., Carver J.R., Zaha V.G. (2021). Cardiovascular Manifestations from Therapeutic Radiation: A Multidisciplinary Expert Consensus Statement From the International Cardio-Oncology Society. JACC CardioOncol..

[24] North B.J., Sinclair D.A. (2012). The intersection between aging and cardiovascular disease. Circ. Res..

[25] López-Otín C., Blasco M.A., Partridge L., Serrano M., Kroemer G. (2013). The hallmarks of aging. Cell.

[26] Madamanchi N.R., Runge M.S. (2007). Mitochondrial dysfunction in atherosclerosis. Circ. Res..

[27] Zamorano J.L., Lancellotti P., Rodriguez Muñoz D., Aboyans V., Asteggiano R., Galderisi M., Habib G., Lenihan D.J., Lip G.Y.H., Lyon A.R. (2016). 2016 ESC Position Paper on cancer treatments and cardiovascular toxicity developed under the auspices of the ESC Committee for Practice Guidelines: The Task Force for cancer treatments and cardiovascular toxicity of the European Society of Cardiology (ESC). Eur. Heart J..

[28] Lakatta E.G., Levy D. (2003). Arterial and cardiac aging: Major shareholders in cardiovascular disease enterprises: Part I: Aging arteries: A “set up” for vascular disease. Circulation.

[29] Loffredo F.S., Nikolova A.P., Pancoast J.R., Lee R.T. (2014). Heart failure with preserved ejection fraction: Molecular pathways of the aging myocardium. Circ. Res..

[30] Mishra S., Kass D.A. (2021). Cellular and molecular pathobiology of heart failure with preserved ejection fraction. Nat. Rev. Cardiol..

[31] Upadhya B., Taffet G.E., Cheng C.P., Kitzman D.W. (2015). Heart failure with preserved ejection fraction in the elderly: Scope of the problem. J. Mol. Cell. Cardiol..

[32] Castiglione V., Gentile F., Ghionzoli N., Chiriacò M., Panichella G., Aimo A., Vergaro G., Giannoni A., Passino C., Emdin M. (2023). Pathophysiological Rationale and Clinical Evidence for Neurohormonal Modulation in Heart Failure with Preserved Ejection Fraction. Card. Fail. Rev..

[33] Lyon A.R., Dent S., Stanway S., Earl H., Brezden-Masley C., Cohen-Solal A., Tocchetti C.G., Moslehi J.J., Groarke J.D., Bergler-Klein J. (2020). Baseline cardiovascular risk assessment in cancer patients scheduled to receive cardiotoxic cancer therapies: A position statement and new risk assessment tools from the Cardio-Oncology Study Group of the Heart Failure Association of the European Society of Cardiology in collaboration with the International Cardio-Oncology Society. Eur. J. Heart Fail..

[34] Fried L.P., Tangen C.M., Walston J., Newman A.B., Hirsch C., Gottdiener J., Seeman T., Tracy R., Kop W.J., Burke G. (2001). Cardiovascular Health Study Collaborative Research Group. Frailty in older adults: Evidence for a phenotype. J. Gerontol. A Biol. Sci. Med. Sci..

[35] Rockwood K., Mitnitski A. (2007). Frailty in relation to the accumulation of deficits. J. Gerontol. A Biol. Sci. Med. Sci..

[36] Clegg A., Young J., Iliffe S., Rikkert M.O., Rockwood K. (2013). Frailty in elderly people. Lancet.

[37] Van den Akker M., Buntinx F., Knottnerus J.A. (1996). Comorbidity or multimorbidity: What’s in a name?. Eur. J. Gen. Pract..

[38] Barnett K., Mercer S.W., Norbury M., Watt G., Wyke S., Guthrie B. (2012). Epidemiology of multimorbidity and implications for health care, research, and medical education: A cross-sectional study. Lancet.

[39] Forman D.E., Maurer M.S., Boyd C., Brindis R., Salive M.E., Horne F.M., Bell S.P., Fulmer T., Reuben D.B., Zieman S. (2018). Multimorbidity in Older Adults with Cardiovascular Disease. J. Am. Coll. Cardiol..

[40] Mohile S.G., Dale W., Somerfield M.R., Schonberg M.A., Boyd C.M., Burhenn P.S., Canin B., Cohen H.J., Holmes H.M., Hopkins J.O. (2018). Practical Assessment and Management of Vulnerabilities in Older Patients Receiving Chemotherapy: ASCO Guideline for Geriatric Oncology. J. Clin. Oncol..

[41] Maher R.L., Hanlon J., Hajjar E.R. (2014). Clinical consequences of polypharmacy in elderly. Expert Opin. Drug Saf..

[42] Gnjidic D., Hilmer S.N., Blyth F.M., Naganathan V., Waite L., Seibel M.J., McLachlan A.J., Cumming R.G., Handelsman D.J., Le Couteur D.G. (2012). Polypharmacy cutoff and outcomes: Five or more medicines were used to identify community-dwelling older men at risk of different adverse outcomes. J. Clin. Epidemiol..

[43] (2015). By the American Geriatrics Society 2015 Beers Criteria Update Expert Panel. American Geriatrics Society 2015 Updated Beers Criteria for Potentially Inappropriate Medication Use in Older Adults. J. Am. Geriatr. Soc..

[44] LeBlanc T.W., McNeil M.J., Kamal A.H., Currow D.C., Abernethy A.P. (2015). Polypharmacy in patients with advanced cancer and the role of medication discontinuation. Lancet Oncol..

[45] World Health Organization (2015). World Report on Ageing and Health.

[46] Swain S.M., Whaley F.S., Ewer M.S. (2003). Congestive heart failure in patients treated with doxorubicin: A retrospective analysis of three trials. Cancer.

[47] Hershman D.L., McBride R.B., Eisenberger A., Tsai W.Y., Grann V.R., Jacobson J.S. (2008). Doxorubicin, cardiac risk factors, and cardiac toxicity in elderly patients with diffuse B-cell non-Hodgkin’s lymphoma. J. Clin. Oncol..

[48] Pinder M.C., Duan Z., Goodwin J.S., Hortobagyi G.N., Giordano S.H. (2007). Congestive heart failure in older women treated with adjuvant anthracycline chemotherapy for breast cancer. J. Clin. Oncol..

[49] Armenian S.H., Lacchetti C., Barac A., Carver J., Constine L.S., Denduluri N., Dent S., Douglas P.S., Durand J.B., Ewer M. (2017). Prevention and Monitoring of Cardiac Dysfunction in Survivors of Adult Cancers: American Society of Clinical Oncology Clinical Practice Guideline. J. Clin. Oncol..

[50] Cardinale D., Colombo A., Bacchiani G., Tedeschi I., Meroni C.A., Veglia F., Civelli M., Lamantia G., Colombo N., Curigliano G. (2015). Early detection of anthracycline cardiotoxicity and improvement with heart failure therapy. Circulation.

[51] Thavendiranathan P., Poulin F., Lim K.D., Plana J.C., Woo A., Marwick T.H. (2014). Use of myocardial strain imaging by echocardiography for the early detection of cardiotoxicity in patients during and after cancer chemotherapy: A systematic review. J. Am. Coll. Cardiol..

[52] Lipshultz S.E., Adams M.J., Colan S.D., Constine L.S., Herman E.H., Hsu D.T., Hudson M.M., Kremer L.C., Landy D.C., Miller T.L. (2013). American Heart Association Congenital Heart Defects Committee of the Council on Cardiovascular Disease in the Young, Council on Basic Cardiovascular Sciences, Council on Cardiovascular and Stroke Nursing, Council on Cardiovascular Radiolo. Long-term cardiovascular toxicity in children, adolescents, and young adults who receive cancer therapy: Pathophysiology, course, monitoring, management, prevention, and research directions: A scientific statement from the American Heart Association. Circulation.

[53] Blum R.H., Carter S.K. (1974). Adriamycin. A new anticancer drug with significant clinical activity. Ann. Intern. Med..

[54] Lichtman S.M., Wildiers H., Chatelut E., Steer C., Budman D., Morrison V.A., Tranchand B., Shapira I., Aapro M., International Society of Geriatric Oncology Chemotherapy Taskforce (2007). International Society of Geriatric Oncology Chemotherapy Taskforce: Evaluation of chemotherapy in older patients—An analysis of the medical literature. J. Clin. Oncol..

[55] Salem J.E., Manouchehri A., Moey M., Lebrun-Vignes B., Bastarache L., Pariente A., Gobert A., Spano J.P., Balko J.M., Bonaca M.P. (2018). Cardiovascular toxicities associated with immune checkpoint inhibitors: An observational, retrospective, pharmacovigilance study. Lancet Oncol..

[56] Lyon A.R., Yousaf N., Battisti N.M.L., Moslehi J., Larkin J. (2018). Immune checkpoint inhibitors and cardiovascular toxicity. Lancet Oncol..

[57] Dolladille C., Ederhy S., Sassier M., Cautela J., Thuny F., Cohen A.A., Fedrizzi S., Chrétien B., Da-Silva A., Plane A.F. (2020). Immune Checkpoint Inhibitor Rechallenge After Immune-Related Adverse Events in Patients with Cancer. JAMA Oncol..

[58] Slamon D.J., Leyland-Jones B., Shak S., Fuchs H., Paton V., Bajamonde A., Fleming T., Eiermann W., Wolter J., Pegram M. (2001). Use of chemotherapy plus a monoclonal antibody against HER2 for metastatic breast cancer that overexpresses HER2. N. Engl. J. Med..

[59] Seidman A., Hudis C., Pierri M.K., Shak S., Paton V., Ashby M., Murphy M., Stewart S.J., Keefe D. (2002). Cardiac dysfunction in the trastuzumab clinical trials experience. J. Clin. Oncol..

[60] Bowles E.J., Wellman R., Feigelson H.S., Onitilo A.A., Freedman A.N., Delate T., Allen L.A., Nekhlyudov L., Goddard K.A., Davis R.L. (2012). Risk of heart failure in breast cancer patients after anthracycline and trastuzumab treatment: A retrospective cohort study. J. Natl. Cancer Inst..

[61] Chavez-MacGregor M., Zhang N., Buchholz T.A., Zhang Y., Niu J., Elting L., Smith B.D., Hortobagyi G.N., Giordano S.H. (2013). Trastuzumab-related cardiotoxicity among older patients with breast cancer. J. Clin. Oncol..

[62] Chen J., Long J.B., Hurria A., Owusu C., Steingart R.M., Gross C.P. (2012). Incidence of heart failure or cardiomyopathy after adjuvant trastuzumab therapy for breast cancer. J. Am. Coll. Cardiol..

[63] Herrmann J., Yang E.H., Iliescu C.A., Cilingiroglu M., Charitakis K., Hakeem A., Toutouzas K., Leesar M.A., Grines C.L., Marmagkiolis K. (2016). Vascular Toxicities of Cancer Therapies: The Old and the New--An Evolving Avenue. Circulation.

[64] Curigliano G., Lenihan D., Fradley M., Ganatra S., Barac A., Blaes A., Herrmann J., Porter C., Lyon A.R., Lancellotti P. (2020). ESMO Guidelines Committee. Electronic address: Clinicalguidelines@esmo.org. Management of cardiac disease in cancer patients throughout oncological treatment: ESMO consensus recommendations. Ann. Oncol..

[65] Campia U., Moslehi J.J., Amiri-Kordestani L., Barac A., Beckman J.A., Chism D.D., Cohen P., Groarke J.D., Herrmann J., Reilly C.M. (2019). Cardio-Oncology: Vascular and Metabolic Perspectives: A Scientific Statement From the American Heart Association. Circulation.

[66] Herrmann J. (2020). Adverse cardiac effects of cancer therapies: Cardiotoxicity and arrhythmias. Nat. Rev. Cardiol..

[67] Attanasio U., Di Sarro E., Tricarico L., Di Lisi D., Armentaro G., Miceli S., Fioretti F., Deidda M., Correale M., Novo G. (2024). Cardiovascular Biomarkers in Cardio-Oncology: Antineoplastic Drug Cardiotoxicity and Beyond. Biomolecules.

[68] Baldassarre L.A., Ganatra S., Lopez-Mattei J., Yang E.H., Zaha V.G., Wong T.C., Ayoub C., DeCara J.M., Dent S., Deswal A. (2022). ACC Cardio-Oncology and the ACC Imaging Councils. Advances in Multimodality Imaging in Cardio-Oncology: JACC State-of-the-Art Review. J. Am. Coll. Cardiol..

[69] Leong D.P., Waliany S., Abdel-Qadir H., Atkins K.M., Neilan T.G., Lang N.N., Liu J.E., Blaes A.H., Mian H.S., Moore H.N. (2024). Cardiovascular Considerations During Cancer Therapy: Gaps in Evidence and JACC: CardioOncology Expert Panel Recommendations. JACC CardioOncol..

[70] Wildiers H., Heeren P., Puts M., Topinkova E., Janssen-Heijnen M.L.G., Extermann M., Falandry C., Artz A., Brain E., Colloca G. (2014). International Society of Geriatric Oncology consensus on geriatric assessment in older patients with cancer. J. Clin. Oncol..

[71] Bellera C.A., Rainfray M., Mathoulin-Pélissier S., Mertens C., Delva F., Fonck M., Soubeyran P.L. (2012). Screening older cancer patients: First evaluation of the G-8 geriatric screening tool. Ann. Oncol..

[72] Rockwood K., Song X., MacKnight C., Bergman H., Hogan D.B., McDowell I., Mitnitski A. (2005). A global clinical measure of fitness and frailty in elderly people. Can. Med. Assoc. J..

[73] Hurria A., Togawa K., Mohile S.G., Owusu C., Klepin H.D., Gross C.P., Lichtman S.M., Gajra A., Bhatia S., Katheria V. (2011). Predicting chemotherapy toxicity in older adults with cancer: A prospective multicenter study. J. Clin. Oncol..

[74] Charlson M.E., Pompei P., Ales K.L., MacKenzie C.R. (1987). A new method of classifying prognostic comorbidity in longitudinal studies. J. Chronic Dis..

[75] Scott J.M., Nilsen T.S., Gupta D., Jones L.W. (2018). Exercise Therapy and Cardiovascular Toxicity in Cancer. Circulation.

[76] Cardinale D., Colombo A., Sandri M.T., Lamantia G., Colombo N., Civelli M., Martinelli G., Veglia F., Fiorentini C., Cipolla C.M. (2006). Prevention of high-dose chemotherapy-induced cardiotoxicity in high-risk patients by angiotensin-converting enzyme inhibition. Circulation.

[77] Guglin M., Krischer J., Tamura R., Fink A., Bello-Matricaria L., McCaskill-Stevens W., Munster P.N. (2019). Randomized Trial of Lisinopril Versus Carvedilol to Prevent Trastuzumab Cardiotoxicity in Patients with Breast Cancer. J. Am. Coll. Cardiol..

[78] Avila M.S., Ayub-Ferreira S.M., de Barros Wanderley J., Das Dores Cruz F., Gonçalves Brandão S.M., Rigaud V.O.C., Higuchi-Dos-Santos M.H., Hajjar L.A., Kalil Filho R., Hoff P.M. (2018). Carvedilol for Prevention of Chemotherapy-Related Cardiotoxicity: The CECCY Trial. J. Am. Coll. Cardiol..

[79] Swain S.M., Whaley F.S., Gerber M.C., Ewer M.S., Bianchine J.R., Gams R.A. (1997). Delayed administration of dexrazoxane provides cardioprotection for patients with advanced breast cancer treated with doxorubicin-containing therapy. J. Clin. Oncol..

[80] Jiang R., Lou L., Shi W., Chen Y., Fu Z., Liu S., Sok T., Li Z., Zhang X., Yang J. (2024). Statins in Mitigating Anticancer Treatment-Related Cardiovascular Disease. Int. J. Mol. Sci..

[81] Cardinale D., Colombo A., Lamantia G., Colombo N., Civelli M., De Giacomi G., Rubino M., Veglia F., Fiorentini C., Cipolla C.M. (2010). Anthracycline-induced cardiomyopathy: Clinical relevance and response to pharmacologic therapy. J. Am. Coll. Cardiol..

[82] Tamburrini G., de Cobos García C., López-Fernández T. (2025). Advances in Cardioprotection Strategies: Focusing on Breast Cancer and Beyond Through Novel Biomarkers and Pharmacological Approaches. Eur. Cardiol..

[83] Neilan T.G., Quinaglia T., Onoue T., Mahmood S.S., Drobni Z.D., Gilman H.K., Smith A., Heemelaar J.C., Brahmbhatt P., Ho J.S. (2023). Atorvastatin for Anthracycline-Associated Cardiac Dysfunction: The STOP-CA Randomized Clinical Trial. JAMA.

[84] Keramida K., Lopez-Fernandez T., Anker M.S., Petrie M.C., Ameri P., Anderson L.J., Bergler-Klein J., Deswal A., Farmakis D., Ibañez B. (2025). Heart failure with preserved ejection fraction in cancer patients and survivors. A scientific statement of the Heart Failure Association of the ESC and the ESC Council of Cardio-Oncology. Eur. J. Heart Fail..

[85] Armenian S.H., Xu L., Ky B., Sun C., Farol L.T., Pal S.K., Douglas P.S., Bhatia S., Chao C. (2016). Cardiovascular Disease Among Survivors of Adult-Onset Cancer: A Community-Based Retrospective Cohort Study. J. Clin. Oncol..

[86] Fitch M.I., Nicoll I., Newton L., Strohschein F.J. (2022). Challenges of Survivorship for Older Adults Diagnosed with Cancer. Curr. Oncol. Rep..

[87] Jaworski C., Mariani J.A., Wheeler G., Kaye D.M. (2013). Cardiac complications of thoracic irradiation. J. Am. Coll. Cardiol..

[88] Denfeld Q.E., Winters-Stone K., Mudd J.O., Gelow J.M., Kurdi S., Lee C.S. (2017). The prevalence of frailty in heart failure: A systematic review and meta-analysis. Int. J. Cardiol..

[89] Soto-Perez-de-Celis E., Li D., Yuan Y., Lau Y.M., Hurria A. (2018). Functional versus chronological age: Geriatric assessments to guide decision making in older patients with cancer. Lancet Oncol..

[90] Kavalieratos D., Gelfman L.P., Tycon L.E., Riegel B., Bekelman D.B., Ikejiani D.Z., Goldstein N., Kimmel S.E., Bakitas M.A., Arnold R.M. (2017). Palliative Care in Heart Failure: Rationale, Evidence, and Future Priorities. J. Am. Coll. Cardiol..

